# FGF-23 Deficiency Impairs Hippocampal-Dependent Cognitive Function

**DOI:** 10.1523/ENEURO.0469-18.2019

**Published:** 2019-03-22

**Authors:** Ann M. Laszczyk, Dailey Nettles, Tate A. Pollock, Stephanie Fox, Melissa L. Garcia, Jing Wang, L. Darryl Quarles, Gwendalyn D. King

**Affiliations:** 1 Department of Neurobiology, University of Alabama at Birmingham, Birmingham, AL 35294; 2 University of Tennessee Medical Health Science Center, Memphis, TN 37290

**Keywords:** post-natal neurogenesis, seizure, synaptic transmission

## Abstract

Fibroblast growth factor receptor (FGFR) and α-Klotho transduce FGF-23 signaling in renal tubules to maintain systemic phosphate/vitamin D homeostasis. Mice deficient for either the ligand, FGF-23, or the co-receptor, Klotho, are phenocopies with both showing rapid and premature development of multiple aging-like abnormalities. Such similarity in phenotype, suggests that FGF-23 and Klotho have co-dependent systemic functions. Recent reports revealed inverse central nervous system (CNS) effects of Klotho deficiency or Klotho overexpression on hippocampal synaptic, neurogenic, and cognitive functions. However, it is unknown whether FGF-23 deficiency effects function of the hippocampus. We report that, similar to Klotho-deficient mice, FGF-23-deficient mice develop dose-dependent, hippocampal-dependent cognitive impairment. However, FGF-23-deficient brains had no gross structural or developmental defects, no change in hippocampal synaptic plasticity, and only minor impairment to postnatal hippocampal neurogenesis. Together, these data provide evidence that FGF-23 deficiency impairs hippocampal-dependent cognition but otherwise results in a brain phenotype that is distinct from the KL-deficient mouse.

## Significance Statement

Although Fibroblast growth factor 23 (FGF-23) is reportedly expressed by the brain, it has no known brain function. In the periphery, kidney transduction of FGF-23 signaling is required for proper phosphate, calcium and vitamin D homeostasis. Recent reports show that Klotho, the obligate FGF-23 co-receptor, is required for multiple hippocampal activities. If Klotho-deficient brain effects are the result of disrupted FGF-23 signaling, similar phenotypes may occur under conditions of FGF-23 deficiency. Herein, studies were undertaken to determine whether FGF-23-deficient mice show impairment of the same hippocampal functions, as a first step toward investigating whether FGF-23 is the mechanism by which Klotho effects brain function. Our data show that FGF-23 is only required for normal hippocampal-dependent cognition suggesting that FGF-23 may affect the brain uniquely.

## Introduction

The fibroblast growth factor 23 (FGF-23) protein is a member of the FGF-19 family of hormonal FGFs. FGF-23 was first characterized as the causal mutation in a case of autosomal dominant hypophosphatemic rickets (ADHR Consortium, 2000). In humans, both increased and decreased expression of FGF-23 causes disease, underscoring the importance of proper phosphate homeostasis for healthy life (Benet-Pagès et al., 2005; Smith et al., 2014). In mouse models, FGF-23 overexpression induces phosphate wasting (Bai et al., 2004; Larsson et al., 2004; Shimada et al., 2004b), while FGF-23 deficiency causes toxic serum accumulation of phosphate and calcium (Shimada et al., 2004a; Sitara et al., 2004; Liu et al., 2006). Although first identified as RNA made in the brain’s ventrolateral thalamic nucleus, thymus, heart, and small intestine (Yamashita et al., 2000), FGF-23 protein is most highly expressed by bone osteoblasts (Riminucci et al., 2003; Mirams et al., 2004; Yoshiko et al., 2007). A remarkable phenotypic similarity between FGF-23-deficient (Shimada et al., 2004a; Sitara et al., 2004; Liu et al., 2006) and Klotho-deficient (Kuro-o et al., 1997) mice led to the discovery that renal FGF-23 signaling through the FGF receptor (FGFR) requires the co-receptor function of α-Klotho (referred to as Klotho throughout; Kurosu et al., 2006).

Although the ventrolateral thalamic nucleus of the nucleus of the central nervous system (CNS) expresses FGF-23 mRNA (Yamashita et al., 2000) and cerebrospinal fluid (CSF) carries FGF-23 protein (Kunert et al., 2017), the functions of FGF-23 in the brain are poorly understood. Hepatic overexpression of FGF-23 causes spatial memory and Schaffer collateral long-term potentiation (LTP) deficits that can be corrected by feeding mice a high phosphate diet (Liu et al., 2011). It is unclear whether overexpression of FGF-23 induces cognitive impairment because of its role in learning and memory, or as an indirect consequence of the peripheral illness caused by high levels of FGF-23. When hippocampal neurons are cultured with FGF-23 supplemented media, less complex neuronal morphology is measured that appears to be FGFR but not Klotho-dependent (Hensel et al., 2016). These *in vitro* results open the possibility of direct and possibly Klotho-independent effects of FGF-23 on hippocampal neurons.

Recent work reported effects of both Klotho deficiency and overexpression on hippocampal postnatal neurogenesis, synaptic plasticity, and cognition (Nagai et al., 2003; Laszczyk et al., 2017; Li et al., 2017). While Klotho deficiency caused premature neurogenic aging, synaptic change, and impaired spatial memory (Laszczyk et al., 2017; Li et al., 2017), Klotho overexpression delays age-related loss of neurogenesis and preserves cognitive function (Dubal et al., 2015; Laszczyk et al., 2017). Although the co-dependent effects of FGF-23 and Klotho are well established for mineral homeostasis, Klotho evolved before the emergence of FGF-23, suggesting that Klotho may have FGF-23-independent actions.

To compare and contrast the *in vivo* effects of FGF-23 and Klotho deficiency on the brain, we measured hippocampal synaptic plasticity, postnatal neurogenesis, and cognitive function of FGF-23-deficient mice. Impairment of hippocampal-dependent cognitive function was not accompanied by synaptic plasticity change and we measured only minor postnatal hippocampal neurogenic niche change. Distinct from the phenotype of the Klotho-deficient and Klotho-overexpressing mice, these results suggest that FGF-23 might function independent of KL within the CNS to impair hippocampal-dependent cognitive functions or else might modulate cognition indirectly, secondary to peripheral illness.

## Materials and Methods

### Animals

Procedures were approved by the University of Alabama at Birmingham Institutional Animal Care and Use Committee in accordance with the recommendations in the Guide for the Care and Use of Laboratory Animals. FGF-23-deficient mice (C57BL/6J) lines were obtained from L. Darryl Quarles (University of Tennessee Health Science Center; Liu et al., 2006). Mice were generated by breeding heterozygotes. All mice were housed with free access to food and water at 26.6°C and humidity maintained above 40%. FGF-23-deficient mice die prematurely from the confluence of dysfunction body-wide caused by hyperphosphatemia (Liu et al., 2006; Kovesdy and Quarles, 2013). Throughout, male and female mice were used. The only exception is in electrophysiology experiments where only male mice were measured. To minimize support FGF-23-deficient mice, Bacon Softies or Nutra-gel (BioServ) was supplied. For lifespan studies, all mice were weighed weekly and monitored at least every 2 d until their natural death or a moribund phenotype was observed. Mice would be declared moribund if found to show combination of physical characteristics including kyphosis, muscle wasting, thinning hair, labored breathing, and decreased movement. When animals were identified as terminal, they were deeply anesthetized and then terminally perfused and fixed. For perfusion, throughout, tissue was collected after transcardial perfusion with Tyrode’s solution (137 mM NaCl, 2.7 mM KCl, 1 mM MgCl_2_, 1.8 mM CaCl_2_, 0.2 mM Na_2_HPO_4_, 12 mM NaHCO_3_, and 5.5 mM glucose) and 4% paraformaldehyde. For slice culture and biochemical applications where fresh tissue was required, mice were anesthetized using isoflurane, once breathing stopped, mice were rapidly decapitated.

### qPCR

RNA was extracted from flash frozen brain or thymus using RNA STAT-60 (Tel-Test Inc.) and cDNA was generated using iScript RT Supermix (Bio-Rad) per manufacturer’s protocols. MRNA was measured by primer/probe duplex qPCR with SsoFast Probes Supermix (Bio-Rad) and Prime time qPCR assays to the mouse 18s ribosomal subunit (Rn18s: Assay ID Mm.PT.49.3175696.g; IDT) and mouse FGF-23 (assay ID Mm.PT.58.14071003; IDT) on a StepOne qPCR system (Applied Biosystems). Fold change relative to adult brain was calculated using the ΔΔCt method (Clinton et al., 2013).

### Electrophysiology

#### Slice preparation

Mice were anesthetized and then sacrificed by decapitation during the 5th week of life. Coronal vibratome sections (400 μm; VT1000S vibratome; Leica) were cut using ice-cold (1–3°C) dissecting solution (120 mM NaCl, 3.5 mM KCl, 0.7 M CaCl_2_, 4.0 mM MgCl_2_, 1.25 mM NaH_2_PO_4_, 26 mM NaHCO_3_, and 10 mM glucose; bubbled with 95% O_2_/5% CO_2_, pH 7.35–7.45). The CA3 region of the hippocampus was removed to prevent recurrent excitation. Using a holding chamber, slices were held at room temperature in dissecting solution and bubbled with 95% O_2_/5% CO_2_ for >1 h before recording. During the recordings, slices were held in a submersion recording chamber perfused with artificial CSF (ACSF; 120 mM NaCl, 3.5 mM KCl, 2.5 mM CaCl_2_, 1.3 mM MgCl_2_, 1.25 mM NaH_2_PO_4_, 26 mM NaHCO_3_, and 10 mM glucose). All experiments were performed at ∼30°C, and experimenters were blind to genotype.

#### Field potential recording

Field EPSPs (fEPSPs) were recorded from stratum radiatum of CA1 using glass micropipettes (2–5 MΩ) filled with ACSF in response to extracellular stimulation of Schaffer Collateral axons by a bipolar tungsten microelectrode (FHC). Stimulation was generated by a Master-8-cp stimulator (A.P.I.) and applied with a BSI-2 biphasic stimulus isolator (BAK Electronics). The initial slope of the fEPSP was used as a measure of synaptic response.

#### Paired-pulse facilitation (PPF)

For measurement of PPF, stimulation was applied as pairs of pulses (interval 50, 100, 150, 200, 250, 300 ms) at 0.1 Hz. The paired-pulse ratio was calculated as fEPSP slope2/fEPSP slope1. ACSF contained picrotoxin (100 μm; Tocris) to block inhibitory GABAA receptors synaptic responses and 100-μm [+]-2-amino-5-phosphonopentanoic acid (APV; Tocris) to block NMDA receptor-mediated currents and prevent postsynaptic short-term plasticity, LTP, and long-term depression.

#### LTP

LTP experiments were performed using an interface chamber (Fine Science Tools). Oxygenated ACSF (95%/5% O_2_/CO_2_; 120 mM NaCl, 2.5 mM KCl, 2 mM CaCl_2_, 1 mM MgCl_2_, 1.25 mM NaH_2_PO_4_, 25 mM NaHCO_3_, and 25 mM glucose) warmed to 30°C (TC-324B temperature controller, Warner Instruments) was perfused into the chamber at 1 ml/min. Electrophysiological traces were amplified (Model 1800 amplifier, A-M Systems), digitized and stored (Digidata models 1322A with Clampex software, Molecular Devices). Extracellular stimuli were administered (Model 2200 stimulus isolator, A-M Systems) on the border of area CA3 and CA1 along the Schaffer-collaterals using enameled, bipolar platinum-tungsten (92%:8%) electrodes. fEPSPs were recorded in stratum radiatum with an ACSF-filled glass recording electrode (1–3 MΩ). The relationship between stimulus intensity and fEPSP slopes over various stimulus intensities (0.5–15 V, 25 nA to 1.5 µA) was used to assess baseline synaptic transmission. High-frequency stimulus-induced LTP was induced by administering three 100-Hz tetani (1-s duration) at an interval of 20 s. Synaptic efficacy was monitored 20 min before and 1–3 h following induction of LTP by recording fEPSPs every 20 s (traces were averaged for every 2-min interval).

### Pentylenetetrazole (PTZ) challenge

PTZ (Sigma) was dissolved in PBS and used at 4 mg/ml. A dose of 60 mg/kg was administered by a single intraperitoneal injection. Immediately thereafter, each mouse was placed in an empty cage and observed for 20 min with simultaneous video recording. Seizure severity was scored by an observer blind to either the mouse line or individual mouse genotype. Scoring is reported as the time following PTZ injection to reach a given stage (latency): (1) immobility/freezing; (2) generalized spasm, tremble or twitch; (3) tail extension to 90°; (4) forelimb clonus; (5) generalized clonic activity; (6) bouncing/running seizure; (7) full tonic extension; (8) death (Roberson et al., 2007, 2011). After 20 min elapsed, all mice were anesthetized with isoflurane and killed by cervical dislocation

### Histologic stains

Fresh dissected tissue was cryoprotected in sucrose, frozen at –80°C, and 10-µm cryostat sections analyzed. Following incubation in 2% Alizarin red solution (pH 4.2), sections were dehydrated with sequential washes through acetone, acetone:xylene, and xylene before mounting in Permount mounting media (Fisher Scientific).

Following perfusion/fixation, as above, serial 30 μm, free-floating coronal sections representing 1/6th of each brain were mounted on gelatinized slides. Following cresyl violet acetate incubation, fixed sections were dH_2_O washed and dehydrated with graded ethanol and xylenes. Volume was estimated in 1/6th of each brain used the Cavalieri estimator and Stereo-Investigator software (–1.22 to –3.88 mm from bregma). When dentate was estimated, total volume, dorsal (–1.22 to –2.18 mm from bregma) and ventral (–2.46 to –3.88 mm from bregma), was measured (Laszczyk et al., 2017).

### Immunohistochemistry (IHC)

Brains were processed through perfusion/fixation, as above. Serial 30 μm, free-floating coronal sections representing 1/6th of each brain were permeabilized in Tris-buffered saline containing Triton X-100 (TBST; 50 mM Tris with 0.9% NaCl, and 0.5% Triton X-100), incubated with 0.3% H_2_O_2_, and blocked with 10% horse serum/TBST. Primary antibodies were incubated in 1% horse serum/TBST for 48 h. Primary antibodies: glial fibrillary acidic protein (GFAP; 1:500, anti-rabbit, Dako Z033429), sex-determining region box 2 (Sox2; 1:100, anti-goat, Santa Cruz Biotechnology, sc-17320), Ki67 (1:500, Abcam ab15580), brain lipid binding protein (BLBP; 1:300, EMD Millipore, ABN14), doublecortin (DCX; 1:50, Santa Cruz Biotechnology, sc-8066, or 1:200 Abcam ab18723), FGF-23 (1:30, R&D Systems, MAB26291; 1:100, Mybiosource MBS2003657), cleaved caspase 3 (1:1000, Cell Signaling 9664), or S100β (1:400, Dako, Z0311). Primary antibody binding was visualized after incubation in fluorescently labeled secondary antibody (Life Technologies). Nuclei were labeled with 4’,6-diamidino-2-phenylindole (DAPI; Life Technologies) and mounted in Prolong Gold anti-fade mounting media (Life Technologies).

#### Quantification

All quantification was performed by genotype blind researchers. Stereology with optical fractionator software (Stereo Investigator version 9, MicroBrightField Inc.) and a Zeiss Axio Imager (Zeiss) microscope fitted with a motorized stage and video camera (AxioCam MRc5, Zeiss; West et al., 1991) was used to estimate sub-granular zone (SGZ) proliferating cells (Ki67 quantifications) every 6th section from –1.34 to –2.10 mm from bregma (dissector height:15-µm with a 5-µm guard zone; fixed counting frame of 80 × 80 μm with a sampling grid size of 75 × 75 μm resulted in 100–200 sites/brain). For all other quantification, the average total number of cells in three sections of dorsal hippocampus is reported (1/6th of each brain processed, –1.34 to –2.10 mm from bregma counted; Gilley et al., 2011; Okamoto et al., 2011). Within this range, maturation stage was defined similar to Plümpe et al., counting 100 cells/brain (Plümpe et al., 2006; Laszczyk et al., 2017).

### Behavior

#### Design

Researchers conducting behavior assays were blind to genotype with mice coded to prevent identification of possible phenotypic differences. FGF-23-deficient mice may be physically distinguished and thus researchers were trained to attend to behavior not mouse body size. Additionally, mice were behavior videos were randomized for before scoring. Mice were habituated to the researcher for three consecutive days. Groups of mice were processed sequentially through open field, spatial novelty, and context-dependent fear conditioning tasks.

#### Open field

On test day mice were placed in the center of the open field apparatus (43 × 43 × 30-cm Plexiglas box). Photo beam detectors quantified all activity for 5 min (ENV-515 software, Med Associates).

#### Object location memory

Testing was conducted consistent with previous protocols (Stefanko et al., 2009; Haettig et al., 2011; Vogel-Ciernia and Wood, 2014; Laszczyk et al., 2017). Objects of the same height: star shaped plastic bath toys and Lego towers were mounted on metal washers to prevent tipping. Objects were compared using naïve mice to ensure objects elicited a similar level of exploratory interest. Experimental mice were habituated to a white Plexiglas testing chamber (39 × 19 × 21 cm); containing only black tape marking the north facing wall and fresh bedding material for two consecutive days. On training day, two identical objects were placed on the same side of the box as the black tape and mice were allowed to freely explore for 10 min (Haettig et al., 2011). Twenty-four hours later, mice were returned to the box for 5 min where one object was displaced to the center, back of the box. Mouse behavior was recorded (TopScan, Clever Sys 2.0). Videos of task performance were manually scored. Interaction was judged to occur if mice were observed to be facing and sniffing within 2 cm of a given object. Mice had to explore each object for at least 5 s during the training phase and 15 s during testing to be included. During training, mice would be excluded if they displayed object/side preference >20%. Three mice were excluded from the final analysis for failure to reach these metrics. For each session, discrimination index was calculated as time spent with the moved object – time spent with the non-moved object/total time × 100 (Vogel-Ciernia and Wood, 2014).

#### Context-dependent fear conditioning

Mice were habituated to the testing room for 2 d. On training day, mice were placed in an operant chamber inside an isolation box (Med Associates) for 5 min. During that time, mice freely explored for the first 2 min. Subsequently, a series of 3, 1 s, 0.5-mA shocks were delivered 1x/min. Mice remained in the chamber for 2 min after the last shock. Twenty-four hours after training, mice were tested by return to the same chamber for 5 min. All training and testing were recorded by automated video tracking system (Med Associates). Percentage of time spent freezing was manually scored by measuring freezing behavior in 5-s intervals.

### Statistics

All data are reported as mean ± SEM. Kaplan–Meier curves were analyzed by log-rank test. Statistical significance was determined using GraphPad Prism (GraphPad version 6) software to perform Student’s *t* test or one-way ANOVA results were determined to be significant if *p* < 0.05 between groups (Table 1).

**Table 1.** Statistical summary

Figure	Data structure	Statistical test	*p* value
1*A*	Dataset with two groups	Log-rank	0.0001 CI: 95%
1*C*	Dataset with two groups	*t* test	0.005 CI: 95%
1*D*, left	Dataset with two groups	*t* test	n.s. CI: 95%
1*D*, right	Dataset with more than two groups	One-way ANOVA	0.0001 CI: 95%
2*A*	Dataset with more than two groups	One-way ANOVA	n.s. CI: 95%
2*B*	Dataset with more than two groups	One-way ANOVA	n.s./0.01 CI: 95%
2*C*	Dataset with more than two groups	One-way ANOVA	n.s./0.005 CI: 95%
2*D*	Dataset with more than two groups	One-way ANOVA	n.s./0.0001 CI: 95%
3*A*	Dataset with two groups	*t* test*	n.s. CI: 95%
3*B*	Dataset with two groups	*t* test*	n.s. CI: 95%
3*C*	Dataset with two groups	*t* test**	n.s. CI: 95%
3*D*	Dataset with two groups	Log-rank	n.s. CI: 95%
3*E*	Dataset with two groups	*t* test*	n.s. CI: 95%
4*A*	Dataset with two groups	*t* test*	n.s. CI: 95%
4*B*	Dataset with two groups	*t* test*	n.s. CI: 95%
4*D*	Dataset with two groups	*t* test*	n.s. CI: 95%
4*E*	Dataset with two groups	*t* test	0.02 CI: 95%
4*F*	Dataset with two groups	*t* test	0.03 CI: 95%
4*G*	Dataset with two groups	*t* test*	0.002 CI: 95%
4*I*	Dataset with two groups	*t* test*	n.s. CI: 95%
4*J*	Dataset with two groups	*t* test*	n.s. CI: 95%

Statistical method and *p* value for all comparisons is detailed by figure, data structure, and statistical test applied. CI indicates confidence interval. When a *p* value was statistically significant (*p* < 0.05), the exact *p* value is reported. Non-significant data are noted as n.s. * denotes that when multiple *t* tests occurred on a single graph, the relevant condition or age was compared between WT and KO mice tested; ** denotes that the KO was compared to the WT at 0, 60, and 90 min.

## Results

### Peripheral effects of FGF-23 deficiency

**Figure 1.**
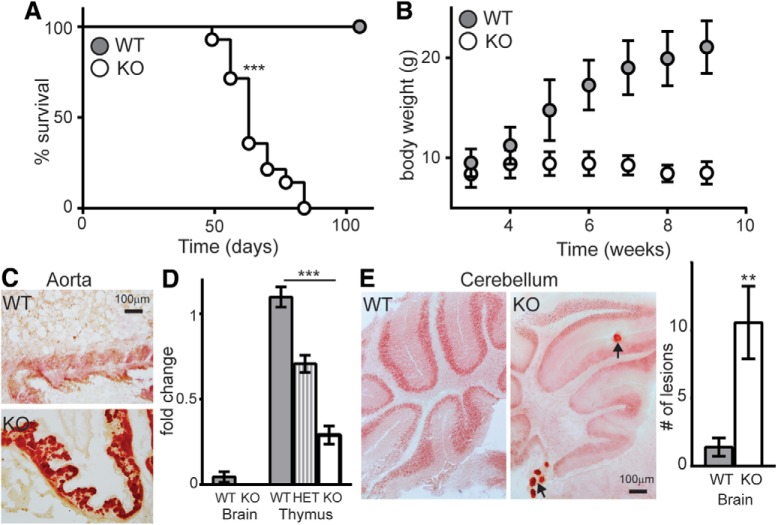
FGF-23-deficient mouse model. ***A***, Kaplan–Meier survival curve of WT and FGF-23-deficient (KO) mouse lifespan showing shortened FGF-23-deficient (KO) mouse lifespan (males and females used; *n* = 10–13/genotype; median survival 63 d, log-rank test ***p < 0.0001). ***B***, During the lifespan study, WT and KO mice were weighed weekly from weaning to death or nine weeks, as relevant. KO mice fail to gain weight after approximately five weeks of age. ***C***, Representative Alizarin red histology of aorta from WT and KO mice. Scale bar represents 100 µm. ***D***, FGF-23 mRNA detected in WT and FGF-23-deficient (heterozygous tissue, HET) adult brain and thymus by ΔΔ C_t_ qPCR (*n* = 5; mean ± SEM; ANOVA, ***p < 0.0001). ***E***, Alizarin red staining of WT and KO brain (cerebellum shown; KO lesions indicated by arrows). Graph shows the average number of calcium lesions (five brains/genotype, average number of lesions counted in three bregma-matched sections; *t* test, **p < 0.005).

FGF-23-deficient mice are reported to live approximately six weeks, show growth retardation and evidence of ectopic calcification (Liu et al., 2006). In our hands, relative to WT littermate controls (WT; C57Bl/6), FGF-23-deficient mice (KO) show a significantly shorter median lifespan of 63 d (Fig. 1*A*). Shortened FGF-23-deficient mouse lifespan was accompanied by growth retardation and prominent ectopic calcification body-wide as detected by Alizarin red staining (Fig. 1*B*,*C*, aorta shown). These results replicate reported FGF-23-deficient mouse peripheral phenotypes (Shimada et al., 2004a; Sitara et al., 2004; Liu et al., 2006).

### FGF-23 deficiency impairs spatial memory

FGF-23 mRNA was originally described in the brain’s ventrolateral thalamic nucleus (Yamashita et al., 2000). Using multiple commercially available antibodies, we were unable to detect WT FGF-23 protein by either IHC or Western blotting of brain tissue. However, quantitative polymerase chain reaction (qPCR) detected low levels of FGF-23 mRNA in WT brain (Fig. 1*D*). Since brain FGF-23 mRNA levels are low, we confirmed dose-dependent FGF-23 deficiency (Yamashita et al., 2000) using a representative FGF-23-expressing peripheral organ, the thymus (Fig. 1*D*). Although FGF-23 is not highly expressed within the brain, FGF-23 mRNA is evident. Prominent ectopic Alizarin red positive, calcium deposition is detected although deposits are smaller and stochastically distributed, relative to peripheral calcification (Fig. 1*E*, cerebellum shown).

**Figure 2.**
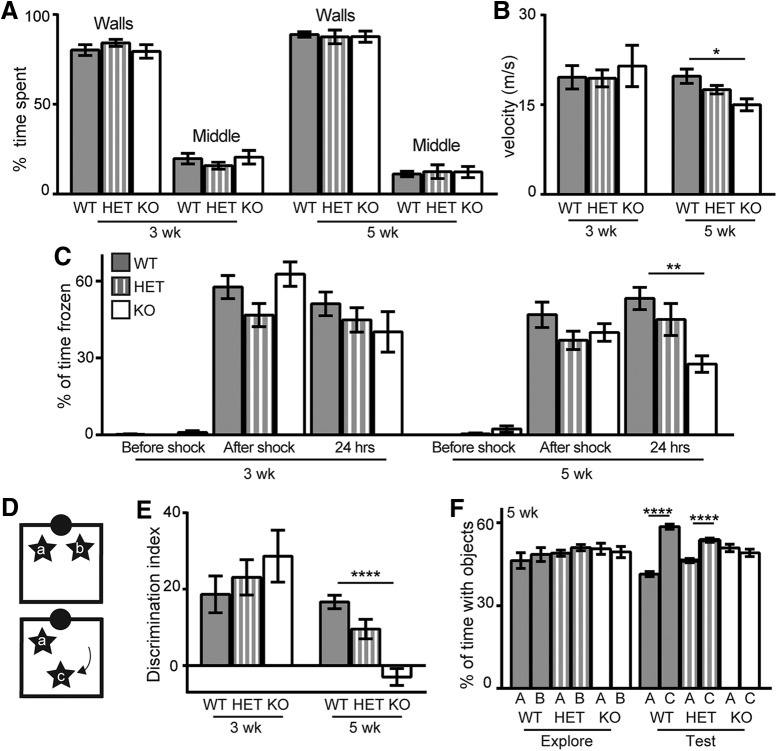
FGF-23 deficiency impairs spatial discrimination. Separate cohorts of WT, HET, and KO mice were measured at three and five weeks of age. ***A***, ***B***, Open field performance % of time spent along the walls or in the middle of the field and velocity (m/s). ***C***, Context-dependent fear conditioning quantified as % of time spent freezing on training day before shock, training day after foot shock, and testing day, 24 h later when returned to the same context. ***D***, Spatial novelty schematic. Identical objects were explored at a set position (positions a and b) and the following day 1 of the objects was moved to a new position (position c). ***E***, ***F***, Spatial novelty scored as the discrimination index [((time with moved – time with non-moved/total time) × 100); three and five weeks] or % of object interaction time spent with each object, each day (five weeks shown; males and females used; *n* = 9–11/genotype; mean ± SEM; ANOVA *p < 0.01, **p < 0.005, ****p < 0.0001).

Klotho deficiency correlates with the rapid onset of hippocampal-dependent cognitive impairment (Nagai et al., 2003; Li et al., 2017). If this effect results from failure of FGF-23 signal transduction through Klotho, FGF-23-deficient mice may be expected show hippocampal-dependent cognitive impairment. Thus, we evaluated hippocampal-dependent spatial memory function in the same behavioral tasks where Klotho-deficient cognitive impairment was reported (Laszczyk et al., 2017). We measured sex-balanced, independent cohorts of mice at three and five weeks of age. Five weeks was selected to ensure mice completed testing before FGF-23-deficient mice naturally begin to die. We evaluated FGF-23-deficient, heterozygous (HET), and WT mice to test for FGF-23 dose-dependent effects on cognition. Inclusion of HET mice also allowed us to ensure that a cognitive phenotype was not exclusively the result of a decline in motor function caused by peripheral disease. FGF-23-deficient mice do not show anxiolytic behavior at three or five weeks of age (Fig. 2*A*). Although indistinguishable at three weeks of age, by five weeks FGF-23-deficient mouse open-field velocity is significantly decreased between groups (Fig. 2*B*). Next, we measured hippocampal-dependent contextual fear conditioning responses. Groups of mice were not different at three weeks of age; however, at five weeks of age, 24 h after training, FGF-23-deficient groups spent less time freezing (Fig. 1*C*). Likewise, when we tested the ability to discriminate closely related contexts using a novel object location memory task (Fig. 1*D*), three-week-old mice are normal but five-week-old FGF-23-deficient mice to not show the normal preference for the moved object (Fig. 1*E*,*F*). Thus similar to what is observed with Klotho deficiency (Laszczyk et al., 2017), FGF-23-deficient mice rapidly develop hippocampal-dependent cognitive impairment evidenced by impaired fear and spatial memory task performance.

### FGF-23 deficiency does not affect hippocampal morphology or Schaffer collateral synaptic plasticity

**Figure 3.**
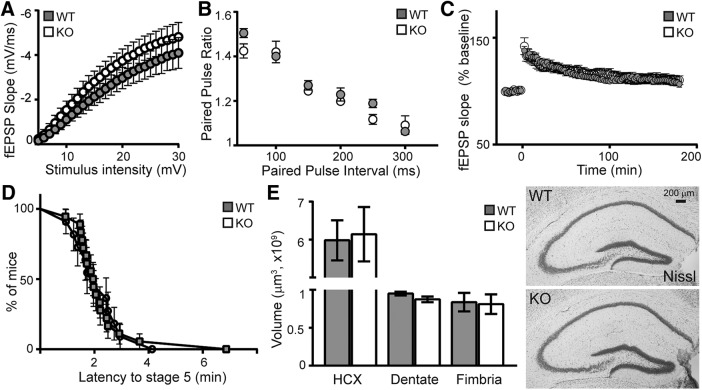
FGF-23 deficiency does not affect gross morphology or synaptic plasticity. WT and KO mice measured at six weeks of age. ***A***, Input-output curves plotted as the initial slopes of the evoked field EPSP (fEPSP; mV/ms) as a function stimulus intensity (mV). No change is measured between genotypes. ***B***, Paired-pulse ratio at 50- to 300-ms intervals showing no differences. ***C***, θ Burst induced LTP measured as the fEPSP over time before and after stimulation is not different between genotypes (male mice only for electrophysiology: *n* = 5–6 mice/genotype, two to four slices per mouse, mean ± SEM; *t* test). ***D***, Mice injected with 60 mg/kg of PTZ were timed for latency to generalized seizure activity. ***E***, Quantification of total hippocampus (HCX), dentate gyrus, and fimbria volumes (–1.22 to –3.88 mm from bregma). Representative WT and KO Nissl stain. Scale bar is 200 µm (males and females used; *n* = 8–10 mice/genotype, mean ± SEM; *t* test).

FGF-23-deficient mouse death is stochastic. To ensure availability of tissue at a common time point, FGF-23-deficient mice and WT controls were killed six weeks after birth. When synaptic plasticity is measured at Schaffer collaterals, Klotho-deficient mice have increased synaptic plasticity with both increased PPF and increased LTP (Li et al., 2017). Using six-week-old FGF-23-deficient slice cultures from male mice, Schaffer collateral synapses show no baseline (input:output), presynaptic (PPF), or postsynaptic (LTP) functional difference (Fig. 3*A–C*). Consistent with normal slice culture results, FGF-23-deficient mice show no change in time to generalized seizure following PTZ injection (Fig. 3*D*). Thus, unlike Klotho-deficient mice (Li et al., 2017), FGF-23 deficiency has no effect on synaptic plasticity at Schaffer collateral synapses.

Neither FGF-23 nor Klotho-deficient mice show disrupted embryonic development. However, adult, Klotho-deficient mice show decreased fimbria volume without a change in the volume of hippocampus or its dentate gyrus (Chen et al., 2013; Laszczyk et al., 2017). Six-week-old FGF-23-deficient mice have identical total hippocampus, dentate gyrus, and fimbria volumes relative to age-matched WT mice (Fig. 3*E*). Unlike Klotho-deficient brains (Chen et al., 2013; Laszczyk et al., 2017), no change in fimbria volume is detected in FGF-23-deficient brain.

### FGF-23 deficiency affects postnatal neurogenesis

**Figure 4.**
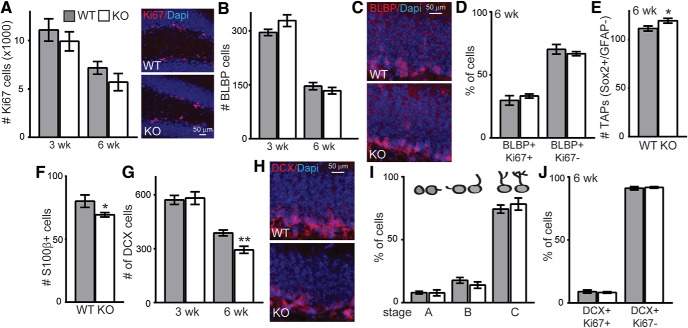
FGF-23 deficiency reduces the number of immature neurons. ***A***, Quantification of all proliferating SGZ cells (Ki67; stereological count) at three and six weeks of age. WT and KO hippocampal neurogenic cell populations as average total cell number across three bregma levels/animal (–1.34 to –2.10 mm) of dorsal hippocampal SGZ. ***B***, Three- and six-week radial-like glial stem cells (BLBP). ***C***, Representative six-week BLBP IHC. ***D***, Percentage of proliferating radial-like glial stem cells (BLBP/Ki67- and BLBP/Ki67+) at six weeks. ***E***, Number of TAPs in six-week-old brain (Sox2+/GFAP-). ***F***, Number of astrocytes (S100β+) in six-week-old SGZ. ***G***, Number of immature neurons (DCX) in three- and six-week-old brain. ***H***, Representative six-week DCX IHC. ***I***, Morphology schematic used to quantify maturation stage of immature neurons at the top of each stage bar graph. Quantification of % of 100 DCX+ immature neurons at each maturation stage. ***J***, Percentage of proliferating immature neurons (DCX/Ki67- and DCX/Ki67+) at six weeks. Scale bars represent 100 or 200 µm; males and females (Lagace et al., 2007) used; *n* = 6–9/genotype, mean ± SEM; *t* test: *p < 0.02, **p < 0.002.

The hippocampal dentate gyrus sub-granular zone (SGZ) is one of the few brain areas with ongoing neurogenesis throughout life (Ming and Song, 2011). Klotho-deficient mice show premature collapse of this neurogenic niche over their shortened lifespan (Laszczyk et al., 2017). Klotho-deficient mice first show decreased SGZ proliferation which proceeds loss of multiple neurogenic cell populations (Laszczyk et al., 2017). To determine whether FGF-23 deficiency impairs postnatal neurogenesis, we measured SGZ progenitor proliferation. FGF-23-deficient brains have the same number of actively proliferating SGZ cells as WT, at both three and six weeks of age (Ki-67; Fig. 4*A*). In postnatal SGZ, generally, radial-like glial precursors divide to produce another radial-like precursor and a cell that then goes on to commit to a neuronal or astrocytic fate (Ming and Song, 2011). Neuronal precursors mature through a series of stages as differential protein expression drives differentiation (Ming and Song, 2011). The neurogenic niche of Klotho model mice is affected at every stage of neuronal differentiation (Laszczyk et al., 2017). However, the total number and number of proliferating FGF-23-deficient SGZ radial-like glial stem-like cells is indistinguishable from WT at either age examined (brain-lipid binding protein, BLBP; Fig. 4*B–D*). We counted a small but significant increase in neuronal committed, transient amplifying progenitors (TAPS; Sox2+/BLBP-; Fig. 4*E*). The number of SGZ-associated astrocytes shows a concomitant significant decrease that could indicate more cells are committing to a neuronal fate (S100β; Fig. 4*F*). However, more mature immature neurons do not corroborate this possibility as the number of FGF-23-deficient immature neurons, while unchanged at three weeks (DCX) is decreased at six weeks (Fig. 4*G*,*H*). We classified the FGF-23-deficient immature neurons by their differentiation stage and found no maturation difference relative to WT (Fig. 4*I*). Similarly, we found no FGF-23-deficient immature neuron proliferation difference (Fig. 4*J*). These results are consistent with FGF-23 deficiency causing an overall reduction in neuronal differentiation without affecting proliferation at any stage. To determine whether increased cell death was occurring, we measured active-caspase 3 expression but it was very low and not different between groups by hippocampal Western blotting or SGZ IHC (data not shown). Thus, unlike neurogenic niche collapse under conditions of Klotho deficiency, FGF-23-deficient neurogenic niches show only minor changes to neurogenesis.

## Discussion

FGF-23 deficiency causes hippocampal-dependent cognitive impairment as measured by impaired fear and object location memory (Fig. 2). These are without an effect on hippocampal synaptic plasticity or network excitability (Fig. 3). Meanwhile, the FGF-23-deficient SGZ shows fewer immature neurons than WT controls (Fig. 4), but the decreased number of immature neurons by six weeks of age is not the result of reduced proliferation, reduced stem cell number, or impaired immature neuron maturation (Fig. 4). Thus, decreased immature neuron number likely represents increased cell death of newly committed neurons (Ryu et al., 2016). The dramatic loss of spatial memory measured by both context-dependent fear conditioning and spatial novelty tasks (Fig. 2) indicates cognitive impairment that is not easily explained by the modest changes we measured in the hippocampus. The FGF-23-deficient brains show ectopic calcification (Fig. 1*E*). Thus, it is possible that hippocampal-dependent cognitive impairment could be the result of an increasingly toxic brain micro-environment caused by FGF-23 deficiency and a resulting lack of normal ion homeostasis.

Although there are reports of CSF circulating FGF-23 (Kunert et al., 2017), we did not detect brain parenchymal FGF-23 protein which may suggest that FGF-23 indirectly affects brain function. As observed with mouse cognitive effects (Liu et al., 2011; Fig. 2), both too much or too little FGF-23 can occur with human illness (ADHR Consortium, 2000; Faul et al., 2011; Singh et al., 2016). Chronic kidney disease patients show increasing serum FGF-23 concentrations as disease worsens (Fliser et al., 2007). These patients can develop cognitive impairment secondary to their systemic kidney disease (Slickers et al., 2007; Sarnak et al., 2013; Hartmann et al., 2015; Hannan et al., 2018) establishing that peripheral renal illness can indirectly impair brain function. Mouse models show that dietary correction of phosphate levels can be sufficient to correct most peripheral symptoms of either FGF-23 signaling deficiency in Klotho-deficient mice (Morishita et al., 2001) or in mice overexpressing FGF-23 (Liu et al., 2011). If an indirect effect of FGF-23 deficiency drives mouse hippocampal-dependent cognitive impairment, correction of phosphate homeostasis may either correct or prevent development of cognitive effects and do so across species impaired by altered FGF-23 levels.

The phenotypic similarity of Klotho and FGF-23-deficient mice (Kuro-o et al., 1997; Shimada et al., 2004b; Sitara et al., 2004) lead to the discovery that FGF-23 signaling is mediated through Klotho/FGFR receptor complexes (Kurosu et al., 2006). While it is clear when FGF-23 function is dependent on Klotho, it remains controversial whether FGF-23 or Klotho also function independently of each other (Fukumoto, 2014). Our studies suggest that Klotho deficiency and FGF-23 deficiency cause different effects on hippocampal synaptic plasticity and postnatal neurogenesis although deficiency of either protein is sufficient to induce cognitive impairment (Fig. 2; Laszczyk et al., 2017). Unlike Klotho deficiency (Laszczyk et al., 2017), FGF-23-deficient mice show no change in synaptic plasticity and very minor effects on adult neurogenesis. This comparison suggests that FGF-23 and Klotho might function independently of one another in the brain as is observed in the heart (Faul et al., 2011). However, in both model systems, conclusive proof that FGF-23 and Klotho function independent of one another are confounded by peripheral illness that could be, either in whole or in part, impacting brain function. Use of cell type-restricted model systems are required for clear molecular dissection of brain-specific FGF-23 and Klotho protein function.

## Synthesis

Reviewing Editor: Margaret McCarthy, Univ of Maryland School of Medicine Decisions are customarily a result of the Reviewing Editor and the peer reviewers coming together and discussing their recommendations until a consensus is reached. When revisions are invited, a fact-based synthesis statement explaining their decision and outlining what is needed to prepare a revision will be listed below. The following reviewer(s) agreed to reveal their identity: Mark Baxter, Pelin Cengiz.

This study looks at the role of fibroblast growth factor 23 (FGF 23) in hippocampal synaptic plasticity, postnatal neurogenesis, and behavioral outcome utilizing the FGF 23 deficient mice. FGF 23 is a potent circulating phosphaturic factor associated with renal phosphate wasting. Effects of FGF 23 on skeleton, phosphate homeostasis, and cardiovascular system have been investigated; however, the effect of FGF 23 on the central nervous system (CNS) is unknown. FGF 23 increases excessively in chronic renal disease resulting in excretion of phosphate. It's function is dependent on the co-receptor Klotho in the kidneys. Both FGF 23 and Klotho have been shown to be expressed in extra-renal tissues. The cognitive problems seen in chronic kidney patients have been linked to FGF 23 overexpression. Recent experimental studies indicate that FGF 23 directly impacts on hippocampal neurons and may thereby impair learning and memory function in chronic kidney disease. Thus, understanding the role of the FGF 23 in CNS will help identify strategies to help patients with chronic kidney disease.

1) In this study, the authors intend to determine whether the Klotho's CNS effects are FGF 23 dependent or independent as they indicate at the end of the introduction. Neither the title nor the results reflect on this question. Their conclusions: 1) Impairment of hippocampal-dependent cognitive function in FGF 23 deficient mice was not accompanied by synaptic plasticity change and 2) Klotho and FGF-23 function independently within the CNS... are not supported by the results and therefore any declarative statements that imply causality should be modified.

2) Critical details such as, sex of the mice and references for the key methodology that is important for reproducibility of the data are missing. Since the FGF 23 protein is not detected in the brain and the mRNA expressions are very low even in WT mice, it is questionable how the conclusions can be based on the role of FGF 23 expression or deficiency. How is the expression of Klotho effected in the absence of FGF 23?

3) It is not very clear how the behavioral tests are performed and how they are interpreted. How is the discrimination ratio calculated? Using common terminology helps reader to understand the test results better. If the discrimination ratio is 50%, mice do not remember the novel location. If it is above 50%, then mice prefer the novel location, if it is less than 50% then mice prefers the familiar location (N/N+F). Do the results suggest that WTs perform similarly as KOs?

4) The authors use a different object location protocol than the previously published protocols. In validated protocols, mice that do not explore novel + familiar location total of 30 min are not included in the analysis (Boulware, M. I., et al. J. Neurosci. 33 (38), 15184-15194, 2013)(Frick, K. M., et al. Behav Neurosci. 117 (6), 1283-1291, 2003). The protocol refences an article (Laszczyk AM, et al.Neurobiol Aging. 2017 Nov;59:41-54) for this test in the discussion, and the methodology for behavioral tests seem exactly the sam eas this reference. Using this protocol, authors concluded that FGF 23 deficiency impairs spatial discrimination (Fig 2D)-conflicting with previous studies. The results show that wild type, hetero and knockout mice at 3 weeks and 5 weeks of life have all discrimination ratios less than 40% (Fig 2D). Calculation of the discrimination ratio is not clear and very confusing. A figure showing the methodology of the object location testing would definitely help understand this protocol.

5) Line 329: Which tests the authors are referring to measure the cognitive function? Open field and fear conditioning tests are used to test anxiety and object location is used to test hippocampal dependent location memory.

6) The image of calcium depositions is not very convincing. Which part of the brain is being shown in Fig 1C?

7) Minor Concerns

-Discussion is very long and unclear. It should discuss only the pertinent findings of the study, too many unrelated facts and unrelated previously published data distracts the attention.

References should be checked - some are missing (e.g. Dubal et al. 2015 cited on line 53)

- line 84: please provide method of euthanasia for mice from which flash frozen brain and thymus tissue is taken

- line 93 - please provide method of anesthesia for mice from which brain slices were prepared

- line 138 - please specify method of blinding given that knockout mice were physically distinguishable from wild-type (as cited in behavioral section) - were same instructions given or were scorers unaware that there were different phenotypes of mice

- line 141 - “anesthetized” instead of “anesthesia”

- line 186 - as for the PTZ seizure scoring, I think it would be helpful to specify whether individuals scoring behavior were aware of the nature of the mice being tested and whether they might expect an impairment in mice that are smaller. The investigators may want to consider whether they could apply an automated scoring method to videos recorded from these experiments (e.g. measuring time mice spend within a zone around an object), or infrared activity monitors in the case of fear conditioning to avoid a requirement for video scoring that could be compromised by experimenter expectations.

- line 256 - should be “deficient”

- line 277 - “affect” should be “effect”

- lines 340, 370 - “independent” should be “independently”

- line 365 - I don't think “commiserate” is the right word here

- Figure 2 caption: ANOVA results should be followed by post hoc tests to determine whether heterozygotes also differ from wild type

- Table 1 statistical summary: instructions to authors specify that “observed power” or a confidence interval on effects should be provided (I favor confidence intervals when these apply)
